# BMC Bioinformatics reviewer acknowledgement 2014

**DOI:** 10.1186/s12859-015-0459-6

**Published:** 2015-03-15

**Authors:** Irene Pala

**Affiliations:** BioMed Central, Floor 6, 236 Gray’s Inn Road, London, WC1X 8HB United Kingdom

## Abstract

The editors of BMC Bioinformatics would like to thank all our reviewers who have contributed their time to the journal in Volume 14 (2013).

**Vida Abedi**

USA

**Mani Abedini**

Australia

**Mohamed Abouelhoda**

Egypt

**Guillaume Achaz**

France

**Jörg Ackermann**

Germany

**Pankaj Agarwal**

USA

**Nima Aghaeepour**

USA

**Daniel Aguilar**

Spain

**Farooq Ahmad**

Pakistan

**Shandar Ahmad**

Japan

**Farzana Ahmed**

Bangladesh

**Marawan Ahmed**

Australia

**Tatsuya Akutsu**

Japan

**Lermine Alban**

France

**Stefan P. Albaum**

Germany

**Danielle Albers**

USA

**Réka Albert**

USA

**Max Alekseyev**

USA

**Boian Alexandrov**

USA

**Ludmil Alexandrov**

UK

**Leonidas Alexopoulos**

Greece

**Jonathan Allen**

USA

**Jens Allmer**

Turkey

**Adam Allred**

USA

**Jonas Almeida**

USA

**Orly Alter**

USA

**Claudia Alteri**

Italy

**Simon Anders**

Germany

**Thomas Levin Andersen**

Denmark

**Wayne Anderson**

USA

**Erick Antezana**

Belgium

**Marianna Apidianaki**

France

**Kazuharu Arakawa**

Japan

**Ana Aransay**

Spain

**Miguel Arenas**

Spain

**Ana Arisi**

Brazil

**Masanori Arita**

Japan

**Nicola Armstrong**

Australia

**Alan Aronson**

USA

**Teresa Attwood**

UK

**Julie Aubert**

France

**Manfred Auer**

USA

**Ferhat Ay**

USA

**Wassim Ayadi**

Tunisia

**Zafer Aydin**

USA

**Kai Christian Bader**

Germany

**Jacob Bælum**

Denmark

**Marc Bailly-Bechet**

France

**Eric Bair**

USA

**Djordje Bajic**

Spain

**Vladimir Bajic**

Saudi Arabia

**Félix Balado**

Ireland

**Petr Baldrian**

Czech Republic

**David Ballard**

USA

**Rajib Bandopadhyay**

India

**Anton Bankevich**

Russian Federation

**Tzachi Bar**

Israel

**Sophie Barbe**

France

**Iros Giacomo Barozzi**

Italy

**Tanya Barrett**

USA

**Harald Barsnes**

Norway

**Baruch Barzel**

Israel

**Frederic Bastian**

Switzerland

**Philippe Batut**

USA

**Michaela Bayerlova**

Germany

**Michael Becich**

USA

**Tim Becker**

Germany

**Khalid Belhajjame**

France

**Riccardo Bellazzi**

Italy

**Christian Bender**

Germany

**Asa Ben-Hur**

USA

**Axel Benner**

Germany

**Chris Benner**

USA

**Panayiotis (Takis) Benos**

USA

**Mikael Benson**

Sweden

**Nir Ben-Tal**

Israel

**Casey Bergman**

UK

**Elsa Bernard**

France

**Ghislain Bidaut**

France

**Patrick Biggs**

New Zealand

**Harald Binder**

Germany

**Silvia Bione**

Italy

**Inanc Birol**

Canada

**Rok Blagus**

Slovenia

**Daniel Blankenberg**

USA

**Michael Blum**

France

**Marten Boetzer**

Netherlands

**Veselka Boeva**

Bulgaria

**Stefan Bonn**

Germany

**Moiz Bootwalla**

USA

**Andrew Bordner**

USA

**Ørnulf Borgan**

Norway

**Erich Bornberg-Bauer**

Germany

**Mark Borodovsky**

USA

**Leonardo Bottolo**

UK

**Alexandre Bouchard**

Canada

**Laura Boucheron**

USA

**Thomas Boudier**

France

**Anne-Laure Boulesteix**

Germany

**Paul Boutros**

Canada

**Frédéric Boyer**

France

**Serdar Bozdag**

USA

**Declan Bradley**

UK

**Keith Bradnam**

USA

**Lauren Bragg**

UK

**Marco Brandizi**

UK

**Amílcar Branquinho**

Portugal

**Emilie Brasset**

France

**Dieter Braun**

Germany

**Nicolas Bray**

USA

**Laurent Brehelin**

France

**Alan Bridge**

Switzerland

**Ryan Brinkman**

Canada

**David Broadhurst**

Canada

**Gudrun Brockmann**

Germany

**Karl Broman**

USA

**Duncan Brown**

USA

**Stefan Bruckner**

Norway

**Eugen Buehler**

USA

**Francesca Buffa**

UK

**Richard Buggs**

UK

**Efstathia Bura**

USA

**Leonid Bystrykh**

Netherlands

**Jiyun Byun**

USA

**Gerard Cagney**

Ireland

**Weidong Cai**

Australia

**José Caldas**

Portugal

**Raffaele Calogero**

Italy

**Erik Cambria**

Singapore

**Robert Campbell**

Canada

**Carlos Cano Gutierrez**

Spain

**Yang Cao**

China

**Salvador Capella-Gutiérrez**

Spain

**J Gregory Caporaso**

USA

**Vincent Carey**

USA

**Marc Carlson**

USA

**Nicole Carnegie**

USA

**Mauricio Carneiro**

USA

**Greg Carter**

USA

**Hannah Carter**

USA

**Antonio Carvajal-Rodriguez**

Spain

**Andre Carvalho**

Brazil

**Benilton Carvalho**

Brazil

**Luis Carvalho**

USA

**Josep Casacuberta**

Spain

**Rita Casadio**

Italy

**Jose Antonio Castellanos Garzon**

Spain

**Michele Ceccarelli**

Italy

**Arnaud Céol**

Italy

**Rengul Cetin-Atalay**

Turkey

**Monica Chagoyen**

Spain

**Hao Chai**

USA

**Mark Chaisson**

USA

**Sutirtha Chakraborty**

USA

**Prabhakar Chalise**

USA

**Raphaël Champeimont**

France

**Cheong Xin Chan**

Australia

**Ramakrishnan Chandrasekaran**

India

**Hsueh-Wei Chang**

Taiwan

**Chia-En Chang**

USA

**Christopher Chang**

USA

**Brad Chapman**

USA

**Wendy Chapman**

USA

**Andrew Chatr-Aryamontri**

UK

**Jagat Singh Chauhan**

India

**Emmanuel Chazard**

France

**Bin Chen**

USA

**Bor-Sen Chen**

Taiwan

**Chia-Yen Chen**

USA

**Guanhua Chen**

USA

**Gary Chen**

USA

**James Chen**

USA

**Jian Chen**

USA

**Jin Chen**

USA

**Ming Chen**

China

**Mengjie Chen**

USA

**Rui Chen**

USA

**Xing Chen**

China

**Yunshun Chen**

Australia

**Zhen Chen**

USA

**Jianlin Cheng**

USA

**Li Cheng**

Singapore

**Yiyu Cheng**

China

**Joshua Cherry**

USA

**Divya Chhabra**

USA

**Nicholas Chia**

USA

**Matteo Chiara**

Italy

**Francesca Chiaromonte**

USA

**Rayan Chikhi**

France

**Greg Chirikjian**

USA

**Ji-Hoon Cho**

USA

**Young-Rae Cho**

USA

**Hoo-Kyun Choi**

South Korea

**Hyungwon Choi**

Singapore

**Sun Shim Choi**

South Korea

**Miew Keen Choong**

Australia

**Uciel Chorostecki**

Argentina

**Panagiotis Chouvardas**

Greece

**Christopher Chute**

USA

**Philipp Cimiano**

Germany

**Angela Ciuffi**

Switzerland

**Lieven Clement**

Belgium

**Jose C. Clemente**

USA

**Olivier Cloarec**

UK

**Peter Clote**

USA

**Bradley Coe**

USA

**Luis Pedro Coelho**

Germany

**Andrew Cohen**

USA

**Roney Coimbra**

Brazil

**Lachlan Coin**

UK

**James Cole**

USA

**Andrew Collins**

UK

**Matteo Comin**

Italy

**Therese Commes**

France

**Ana Conesa**

Spain

**Karen Conneely**

USA

**Lee Cooper**

USA

**Laurel Copeland**

USA

**Dario Copetti**

USA

**Juan Corchado**

Spain

**Rebecca Cordell**

UK

**Francesca Cordero**

Italy

**Thorben Cordes**

Netherlands

**Claudia Coronnello**

Italy

**Hector Corrada Bravo**

USA

**Ivan Costa**

Germany

**Rafael Costa**

Portugal

**Jasmin Coulombe-Huntington**

Canada

**Francisco Couto**

Portugal

**David Craik**

Australia

**Mark Craven**

USA

**Peter Csermely**

Hungary

**Alvaro Cuadros-Inostroza**

Germany

**Engin Cukuroglu**

Turkey

**Hong-Jie Dai**

Taiwan

**Timothy Daley**

USA

**Thomas Dandekar**

Germany

**Ludmila Danilova**

USA

**Laurent Dardenne**

Brazil

**Melissa Davis**

Australia

**Ramana Davuluri**

USA

**Peter Dawyndt**

Belgium

**Alexandre De Brevern**

France

**Joao Pedro De Magalhaes**

UK

**Jan De Neve**

Belgium

**Tulio De Oliveira**

South Africa

**Katleen De Preter**

Belgium

**Yves Dehouck**

Belgium

**Cristian Del Fabbro**

Italy

**Gilbert Deleage**

France

**Nicolas Delhomme**

Sweden

**Doulaye Dembélé**

France

**Jingyuan Deng**

USA

**Sebastian Deorowicz**

Poland

**Christophe Dessimoz**

UK

**Samuel Deutsch**

USA

**Marcella Devoto**

USA

**Eva Dhondt**

France

**Barbara Di Camillo**

Italy

**Massimo Di Giulio**

Italy

**Rebecca Dickstein**

USA

**Ferran Diego Andilla**

Germany

**Christoph Dieterich**

Germany

**Marcel Dinger**

Australia

**Huy Dinh**

USA

**Irina Dinu**

Canada

**Marcus Dittrich**

Germany

**Ugur Dogrusoz**

Turkey

**Frank Dondelinger**

Netherlands

**Suomeng Dong**

UK

**Karin Dorman**

USA

**Bryan Downie**

Germany

**Johannes Dröge**

Germany

**Tom Druet**

Belgium

**Roshan D’Souza**

USA

**Xiuquan Du**

China

**Pufeng Du**

China

**Junbo Duan**

USA

**Inna Dubchak**

USA

**Konsta Duesing**

Australia

**Alexandre Dufour**

France

**Michel Dumontier**

Canada

**Ritaban Dutta**

Australia

**Daniel Dvorkin**

USA

**Timothy Ebbels**

UK

**Afshin Ebrahimpour**

Malaysia

**Robert Edgar**

USA

**Arwyn Edwards**

UK

**Todd Edwards**

USA

**Ralf Eggeling**

Germany

**Marinus Eijkemans**

Netherlands

**Anders Eklund**

USA

**Aron Eklund**

Denmark

**Mohamed Elasri**

USA

**Mohammed El-Kebir**

Netherlands

**Yasser El-Manzalawy**

Egypt

**Richard Emes**

UK

**Frank Emmert-Streib**

UK

**Kerry Emslie**

Australia

**Toshinori Endo**

Japan

**Christine Eng**

Singapore

**Kristof Engelen**

Belgium

**Hatice Billur Engin**

USA

**Fabian Erdel**

Germany

**Evangelos Evangelou**

Greece

**Eran Eyal**

Israel

**Marc Facciotti**

USA

**James Faeder**

USA

**Francois Fages**

France

**Xiaodan Fan**

Hong Kong

**Lin Fang**

China

**Jianwen Fang**

USA

**Xin Fang**

USA

**Zhide Fang**

USA

**Maha Farhat**

USA

**Piero Fariselli**

Italy

**Illes J. Farkas**

Hungary

**Melanie Febrer**

UK

**Maria Federico**

Italy

**Dmitry Fedorov**

USA

**K. Anton Feenstra**

Netherlands

**Soheil Feizi**

USA

**Howard Feldman**

Canada

**F. Alex Feltus**

USA

**Zhixing Feng**

USA

**Tim Fennell**

USA

**Jerome Feret**

France

**Xose Fernandez**

UK

**Evandro Ferrada**

USA

**Natosha Finley**

USA

**Stephen Fiore**

USA

**Larry Mark Fisher**

UK

**Jason Flannick**

USA

**Avrilia Floratou**

USA

**Darren Flower**

UK

**Christopher Flowers**

USA

**Friedrich Foerster**

Germany

**Nuno Fonseca**

UK

**Jean-Fred Fontaine**

Germany

**Wolfgang Förstner**

Germany

**Jan Fostier**

Belgium

**Octavio Franco**

Brazil

**Ashley Franks**

Australia

**Christian Frech**

Canada

**David Fredman**

Austria

**Leon French**

Canada

**Kenneth G Frey**

USA

**Caroline Friedel**

Germany

**Mikko Frilander**

Finland

**Holger Fröhlich**

Germany

**Florian Frommlet**

Austria

**Yan Fu**

China

**Georg Fuellen**

Germany

**Matthew Fullmer**

USA

**Melissa Fullwood**

Singapore

**Toni Gabaldon**

Spain

**Jill Gallaher**

USA

**Giorgio Galli**

USA

**Oxana Galzitskaya**

Russian Federation

**Philippe Gambette**

France

**Jiajnun Gan**

USA

**Angel Garcia**

USA

**Juan Antonio Garcia**

Austria

**Juan M Garcia-Gomez**

Spain

**Vincent Gardeux**

USA

**Shea Gardner**

USA

**Pascale Gaudet**

Switzerland

**Nils Gehlenborg**

USA

**Giulio Genovese**

USA

**Wolfgang Gerlach**

USA

**Damian Gessler**

USA

**Jana Gevertz**

USA

**Mahmoud Ghandi**

USA

**Joyee Ghosh**

USA

**John Gibbons**

USA

**Yolanda Gil**

USA

**Walter Gilks**

UK

**Alessandro Giuliani**

Italy

**Julien Gobeill**

France

**Carsten Goerg**

USA

**Andre Gohr**

Germany

**Aaron Golden**

USA

**Andres Gomez**

Spain

**David Gomez-Cabrero**

Spain

**Wuming Gong**

USA

**Abel Gonzalez-Perez**

Spain

**Balasubramanian Gopal**

India

**Fabio Gori**

Netherlands

**Naohisa Goto**

Japan

**Osamu Gotoh**

Japan

**Assaf Gottlieb**

USA

**Julian Gough**

UK

**Joel Graber**

USA

**Manfred Grabherr**

USA

**Jan Grau**

Germany

**Paul Greenfield**

Australia

**Darren Griffin**

UK

**Dominik Grimm**

Germany

**Martien Groenen**

Netherlands

**Michael Gromiha**

India

**Ana Rita Grosso**

Portugal

**Mathieu Groussin**

France

**Jin Gu**

China

**Zuguang Gu**

Germany

**Alain Guenoche**

France

**Ulrich L Guenther**

UK

**Leandro Guerrero**

South Africa

**Vincent Guillemot**

France

**Stephane Guindon**

New Zealand

**Justin Guinney**

USA

**Rudiyanto Gunawan**

Switzerland

**Emre Guney**

USA

**An-Yuan Guo**

China

**Bhagwati Gupta**

Canada

**Attila Gursoy**

Turkey

**Albert Guskov**

Netherlands

**Pietro Hiram Guzzi**

Italy

**Frederik Gwinner**

France

**Gavin Ha**

USA

**Brian Haas**

USA

**Vanja Haberle**

Norway

**Tsuyoshi Hachiya**

Japan

**Markus Hafner**

USA

**Paul Hagerman**

USA

**Michiaki Hamada**

Japan

**Donald Hamelberg**

USA

**Carol Hamilton**

USA

**Nicholas Hamilton**

Australia

**Jie Han**

Canada

**Mira Han**

USA

**Thomas James Hardcastle**

UK

**Henk Harkema**

UK

**Sarah Harris**

UK

**Roger Harrison**

USA

**Benjamin Harshfield**

USA

**Steven Hart**

USA

**Soha Hassoun**

USA

**Bernhard Haubold**

Germany

**David Haughton**

Ireland

**Morihiro Hayashida**

Japan

**Tao He**

USA

**Ping-An He**

China

**Qing-Yu He**

China

**Zengyou He**

China

**Lenwood Heath**

USA

**Jayne Hehir-Kwa**

Netherlands

**Dominik Heider**

Germany

**Merja Heinäniemi**

Finland

**Michael Henson**

USA

**Enrique Hernandez-Lemus**

Mexico

**Carl Herrmann**

Germany

**Ralf Herwig**

Germany

**Yulia Hicks**

UK

**Alicia Hidalgo**

UK

**Falk Hildebrand**

Germany

**Christopher Hill**

USA

**Steven Hill**

UK

**Lynette Hirschman**

USA

**Harry Hochheiser**

USA

**Christian Hoener Zu Siederdissen**

Austria

**Ralf Hofestädt**

Germany

**Katharina Jasmin Hoff**

Germany

**Peter Holmans**

UK

**Manuel Holtgrewe**

Germany

**Hao Hongwei**

China

**Stefan Hoops**

USA

**Fereydoun Hormozdiari**

USA

**Peter Hornbeck**

USA

**Parsa Hosseini**

USA

**Yong Hou**

China

**Lin Hou**

USA

**E. Andres Houseman**

USA

**Adina Howe**

USA

**Jeremy Hsu**

USA

**Gangqing Hu**

USA

**Jianhua Hu**

USA

**Zihua Hu**

USA

**Guang-Bin Huang**

Singapore

**Junzhou Huang**

USA

**Liang-Tsung Huang**

Taiwan

**R. Stephanie Huang**

USA

**Jaime Huerta-Cepas**

Germany

**Jui-Hung Hung**

Taiwan

**Rachael Huntley**

UK

**Mark Hurle**

USA

**Flavia Huygens**

Australia

**Torgeir R. Hvidsten**

Norway

**Michael Imelfort**

Australia

**Francesco Iorio**

UK

**Anders Isaksson**

Sweden

**Musarat Ishaq**

Australia

**Jon Ison**

UK

**Kimihito Ito**

Japan

**Sergey Ivanov**

USA

**Hosna Jabbari**

Canada

**Shaun Jackman**

Canada

**Anders Jacobsen**

Denmark

**Aruna Jammalamadaka**

USA

**Sarath Chandra Janga**

USA

**Silke Janitza**

Germany

**Stefan Janssen**

Germany

**Marine Jeanmougin**

France

**Lars S Jermiin**

Australia

**Robert Jernigan**

USA

**Guoli Ji**

China

**Hongkai Ji**

USA

**Pengsheng Ji**

USA

**Dingfeng Jiang**

USA

**Duo Jiang**

USA

**Hui Jiang**

USA

**Rui Jiang**

China

**Zhenran Jiang**

China

**Radu Jianu**

USA

**Rafael Jimenez**

UK

**Antonio Jos & Eacute; Jimeno Yepes**

USA

**Guo Jinchao**

China

**Andrzej Joachimiak**

USA

**W. Evan Johnson**

USA

**Inge Jonassen**

Norway

**Siddhartha Jonnalagadda**

USA

**Alark Joshi**

USA

**Daniel Jost**

France

**Fabien Jourdan**

France

**David Juan**

Spain

**Hsueh-Fen Juan**

Taiwan

**Ivan Junier**

Spain

**Thomas Junier**

Switzerland

**Yannis Kalaidzidis**

Germany

**Hyun Min Kang**

USA

**Aditi Kanhere**

UK

**Noam Kaplan**

USA

**Ezgi Karaca**

Germany

**Tobias Karakach**

Canada

**Konrad Karczewski**

USA

**Donna Karolchik**

USA

**Yuki Kato**

Japan

**Lee Katz**

USA

**Enver Kayaaslan**

Turkey

**Kevin Keegan**

USA

**Birte Kehr**

Germany

**Patrick Kemmeren**

Netherlands

**Jessie Kennedy**

UK

**Andreas Kerren**

Sweden

**Ozlem Keskin**

Turkey

**Can Kesmir**

Netherlands

**Hans Kestler**

Germany

**Zia Khan**

USA

**Tsung Fei Khang**

Malaysia

**Sawsan Khuri**

USA

**Rami Khushaba**

Australia

**Adiqa Kausar Kiani**

USA

**Halil Kilicoglu**

USA

**Eun-Youn Kim**

Korea, South

**Jin-Dong Kim**

Japan

**Jung-Jae Kim**

Singapore

**Kyoung Heon Kim**

Korea, South

**Seongho Kim**

USA

**Yoo-Ah Kim**

USA

**Sun Kim**

USA

**Wonkuk Kim**

South Korea

**Robert Kincaid**

USA

**Carl Kingsford**

USA

**Alexandros Kiparissides**

Switzerland

**Toralf Kirsten**

Germany

**Hisanori Kiryu**

Japan

**Andrey Kislyuk**

USA

**Teemu Kivioja**

Finland

**Gunnar W. Klau**

Netherlands

**Bernd Klaus**

Germany

**Hans-Ulrich Klein**

Germany

**Judith Klein-Seetharaman**

USA

**Konstantin Klenin**

Germany

**Jacob Koehler**

USA

**Devin Koestler**

USA

**Daria Kokh**

Germany

**Andrzej Kolinski**

Poland

**Martin Kollmar**

Germany

**Ravikumar Komandur Elayavilli**

USA

**Christian Komusiewicz**

Germany

**Yong Kong**

USA

**Nobuaki Kono**

Japan

**Imhoi Koo**

USA

**Tamas Korcsmaros**

Hungary

**Sergey Koren**

USA

**Sandhya Kortagere**

USA

**Sophia Kossida**

Greece

**Johannes Köster**

Germany

**Frank Kramer**

Germany

**Janos Kriston-Vizi**

Singapore

**Martin Krzywinski**

Canada

**Maik Kschischo**

Germany

**Hung-Chih Ku**

Taiwan

**Ivan Kulakovskiy**

Russian Federation

**Anshul Kundaje**

USA

**Anne Kupczok**

Austria

**Shigehiro Kuraku**

Japan

**Lukasz Kurgan**

Canada

**Yutaka Kuroda**

Japan

**Ludovic Kurunczi**

Romania

**Guray Kuzu**

Turkey

**Hoi Shan Kwan**

Hong Kong

**Dilraj Lama**

Singapore

**Claudia Lamina**

Austria

**Jenna Lang**

USA

**Philipp Lange**

Canada

**Nicholas Larson**

USA

**Anders Larsson**

Sweden

**Roman Laskowski**

UK

**Jessica Lasky-Su**

USA

**Timo Lassmann**

Japan

**Sneh Lata**

USA

**Augustinus Laude**

Singapore

**Dominique Lavenier**

France

**Richard Lavery**

France

**Charity Law**

Switzerland

**Yan Nei Law**

Singapore

**Cathy Lawson**

USA

**Vladimir Lazarevic**

Switzerland

**Quan Le**

Ireland

**Kim-Anh Lê Cao**

Australia

**Robert Leaman**

USA

**Marcus Lechner**

Germany

**Dong-Yup Lee**

Singapore

**Tzong-Yi Lee**

Taiwan

**Heewook Lee**

USA

**Richard Lee**

USA

**Su-In Lee**

USA

**Young Ji Lee**

USA

**Christophe Lefevre**

Australia

**Andreas Leha**

Germany

**Rainer Lehtonen**

Finland

**Chris-Andre Leimeister**

Germany

**Forian Leitner**

Spain

**Heike Leitte**

Germany

**Louis-Philippe Lemieux Perreault**

Canada

**Ning Leng**

USA

**Hans-Peter Lenhof**

Germany

**Ulf Leser**

Germany

**Ks Leung**

Hong Kong

**Nicholas Lewin-Koh**

USA

**Alexander Lex**

USA

**Biao Li**

USA

**Cheng Li**

USA

**Gengxin Li**

USA

**Hongzhe Li**

USA

**Jieyue Li**

USA

**Jinyan Li**

Australia

**Jingyi Jessica Li**

USA

**Kelvin Li**

USA

**Chun Li**

China

**Jiao Li**

China

**Ming Li**

USA

**Xiang Li**

USA

**Xia Li**

China

**Mingyao Li**

USA

**Nan Li**

China

**Qi Li**

USA

**Qiyuan Li**

USA

**Xuehui Li**

USA

**Yali Li**

USA

**Yanen Li**

USA

**Yang Li**

USA

**Yunfei Li**

USA

**Han Liang**

USA

**Jianming Liang**

USA

**Chun Liang**

USA

**Aristidis Likas**

Greece

**Ivan Limongelli**

Italy

**Chun-Yuan Lin**

Taiwan

**Dongdong Lin**

USA

**Hao Lin**

China

**Na Lin**

China

**Lynn Lin**

USA

**Ying-Chih Lin**

Taiwan

**Michael Linderman**

USA

**James Lindsay**

USA

**Thomas Lingner**

Germany

**Christoph Lippert**

USA

**Stefano Lise**

UK

**Chung-Feng Liu**

Taiwan

**Jin Liu**

USA

**Li Liu**

USA

**Xiaowen Liu**

USA

**Yu-Shen Liu**

China

**Tao Liu**

USA

**Xiaoming Liu**

USA

**Yaping Liu**

USA

**Yi Liu**

China

**Zhandong Liu**

USA

**Zhi-Ping Liu**

China

**Vebjorn Ljosa**

USA

**Jason Lloyd-Price**

Finland

**Alexander Lobkovsky**

USA

**Martin Loewer**

Germany

**Po-Ru Loh**

USA

**Nicolas Lomenie**

France

**Stefano Lonardi**

USA

**Brian Long**

USA

**Lit-Hsin Loo**

Singapore

**Mario Looso**

Germany

**Joseph Loparo**

USA

**Anne Lopes**

France

**Guillermo Lopez Campos**

Australia

**Daniel López López**

Spain

**Doug Lorenz**

USA

**Xinghua Lou**

USA

**David Lovell**

Australia

**Ari Löytynoja**

UK

**Chuan Lu**

UK

**Hong-Mei Lu**

China

**Le Lu**

USA

**Hong Lu**

USA

**Zhiyong Lu**

USA

**Ira Lubin**

USA

**Suryani Lukman**

United Arab Emirates

**Sajitha Lulu**

India

**Ole Lund**

Denmark

**Steven Lund**

USA

**Rui Luo**

USA

**Yves Lussier**

USA

**Andrew Lynn**

India

**Lan Ma**

USA

**Li Ma**

USA

**Jianxin Ma**

USA

**Avi Ma’Ayan**

USA

**Jose Machado**

Portugal

**Raghu Machiraju**

USA

**Dan Maclean**

UK

**Tom Madden**

USA

**Mallur Madhusudhan**

India

**Huda A. Maghawry**

Egypt

**Despoina Magka**

UK

**Eamonn Maguire**

UK

**Frédéric Mahé**

Germany

**Shaun Mahony**

USA

**Pradipta Maji**

India

**Man-Wai Mak**

Hong Kong

**Wojciech Makalowski**

Germany

**Lars Malmström**

Switzerland

**James Malone**

UK

**Nicholas Mancuso**

USA

**Ulrich Mansmann**

Germany

**Yuqing Mao**

USA

**Jessica Mar**

USA

**Bickle Marc**

Germany

**Luigi Marchionni**

USA

**Aron Marchler-Bauer**

USA

**Edward M. Marcotte**

USA

**Pablo Marin Garcia**

Spain

**Georgi Marinov**

USA

**David Marshall**

UK

**Alan Marshall**

USA

**Marie-Claude Marsolier-Kergoat**

France

**Pier Luigi Martelli**

Italy

**Marcel Martin**

Germany

**Maryann Martone**

USA

**Pekka Marttinen**

Finland

**Manja Marz**

Germany

**Anthony Mathelier**

Canada

**David Mathews**

USA

**Venkat Mathura**

USA

**Sérgio Matos**

Portugal

**Norio Matsushima**

Japan

**Markus Maucher**

Germany

**Giancarlo Mauri**

Italy

**Anoop Mayampurath**

USA

**Raja Mazumder**

USA

**Matthew Nicholson Mccall**

USA

**Davis Mccarthy**

UK

**Jason Mcdermott**

USA

**Steve Mckeever**

Sweden

**Brett Mckinney**

USA

**Claudine Medigue**

France

**Molly Megraw**

USA

**Peter Meinicke**

Germany

**Volodymyr Melnykov**

USA

**Daniel Melters**

USA

**Vesna Memisevic**

USA

**Joerg Menche**

Hungary

**Luis Mendoza**

USA

**Mark Menor**

USA

**Tayeb Merabti**

France

**Enrique Merino**

Mexico

**Rainer Merkl**

Germany

**Bart Mertens**

Netherlands

**Gábor Mészáros**

Austria

**Folker Meyer**

USA

**Massimo Mezzavilla**

Italy

**Gos Micklem**

UK

**Martin Middendorf**

Germany

**Jeffrey Miecznikowski**

USA

**Tijana Milenkovic**

USA

**Andrew Millar**

UK

**Julie Mitchell**

USA

**Faheem Mitha**

USA

**Balraj Mittal**

India

**Sayaka Miura**

USA

**Makoto Miwa**

UK

**Qianxing Mo**

USA

**Iain Moal**

Spain

**Shahin Mohammadi**

USA

**Debasisa Mohanty**

India

**Mark Moll**

USA

**Torsten Möller**

Austria

**Stephen Montgomery**

USA

**Jason Moore**

USA

**Roser Morante**

Belgium

**Fernando Moreno-Herrero**

Spain

**Matthew Morgan**

Australia

**Burkhard Morgenstern**

Germany

**Etsuko Moriyama**

USA

**Robert Morris**

USA

**John Morris**

USA

**David Morrison**

Sweden

**Roberto Mosca**

Germany

**Ivan Moszer**

France

**Julien Mozziconacci**

France

**Arne Mueller**

Germany

**Neelanjan Mukherjee**

USA

**Nandita Mukhopdhyay**

USA

**Francesca Mulas**

Italy

**Tobias Müller**

Germany

**Tamara Munzner**

Canada

**Alexey Murzin**

UK

**Jose Nacher**

Japan

**Nozomi Nagano**

Japan

**Sridevi Nagaraja**

USA

**Niranjan Nagarajan**

USA

**Radhakrishnan Nagarajan**

USA

**Masao Nagasaki**

Japan

**Sigve Nakken**

Norway

**Christine Nardini**

China

**Darren Natale**

USA

**Alejandro Nato**

USA

**Tim W Nattkemper**

Germany

**Roy Navon**

USA

**Russell Neches**

USA

**Alexander J. Nederbragt**

Norway

**Sven Nelander**

Sweden

**Rex Nelson**

USA

**William Nes**

USA

**Pierre Neuvial**

France

**Heiko Neuweger**

Germany

**Aurelie Neveol**

France

**Mariana Neves**

Germany

**Charlotte Ng**

USA

**Shu-Kay Angus Ng**

Australia

**Hannah Nicholas**

Australia

**Pierre Florian Nicolas**

France

**Cydney Nielsen**

Canada

**Henrik Nielsen**

Denmark

**Yoshihito Niimura**

Japan

**Kang Ning**

China

**Motohide Nishio**

Japan

**Corey Nislow**

Canada

**Intawat Nookaew**

Sweden

**Yahaya M. Normi**

Malaysia

**Michael North**

USA

**Francisco Novo**

Spain

**Tom Nye**

UK

**Ann Oberg**

USA

**Sean O’Donoghue**

Germany

**Layla Oesper**

USA

**Sunghee Oh**

USA

**Michal Okoniewski**

Switzerland

**Baldo Oliva**

Spain

**Jose A. Olivas**

Spain

**Daniel Oliveira**

Brazil

**Jose L. Oliver**

Spain

**Noah Ollikainen**

USA

**Michael O’Neill**

USA

**Raydonal Ospina**

Brazil

**Joji Otaki**

Japan

**Ross Overbeek**

USA

**Casey Overby**

USA

**Lex Overmars**

Netherlands

**Lassi Paavolainen**

Finland

**Roland A. Pache**

USA

**Gary Packard**

USA

**Justin Page**

USA

**Robert Page**

USA

**Stefano Maria Pagnotta**

Italy

**Tun-Wen Pai**

Taiwan

**Krishna Palaniappan**

USA

**Wei Pan**

USA

**Vera Pancaldi**

Spain

**Anna Panchenko**

USA

**Binay Panda**

India

**Alessandro Pandini**

UK

**Herb Pang**

Hong Kong

**Natalya Panteleyeva**

USA

**Dimitris Papamichail**

USA

**Jason Papin**

USA

**Francesco Pappalardo**

Italy

**Maria Paraskevopoulou**

Greece

**Changyi Park**

South Korea

**Craig Parker**

USA

**Helen Parkinson**

UK

**Christian Partl**

Austria

**Bogdan Pasaniuc**

USA

**Kaustubh Patil**

Germany

**Ellis Patrick**

Australia

**Rob Patro**

USA

**Grégory Paul**

Switzerland

**Sandip Paul**

India

**Florian Pauler**

Austria

**Stefan Paulus**

Germany

**Paul Pavlidis**

Canada

**Florencio Pazos**

Spain

**Jean Peccoud**

USA

**Shyamal Peddada**

USA

**Tiago Peixoto**

Germany

**Hanchuan Peng**

USA

**Bethany Percha**

USA

**Paulino Pérez**

Mexico

**Jose E Perez-Ortin**

Spain

**Yasset Perez-Riverol**

UK

**Andy Perkins**

USA

**Dragan Perovic**

Germany

**Anton Persikov**

USA

**Bengt Persson**

Sweden

**Catia Pesquita**

Portugal

**Linda Petzold**

USA

**Jonathan Pevsner**

USA

**Son Pham**

USA

**Tuan Pham**

Japan

**Adam Phillippy**

USA

**Angkoon Phinyomark**

Thailand

**Stephen Piccolo**

USA

**Surapong Pinitglang**

Thailand

**David Pinto**

Mexico

**Douglas Pires**

Brazil

**Rosario Michael Piro**

Germany

**Mehdi Pirooznia**

USA

**Clara Pizzuti**

Italy

**Francisco J Planes**

Spain

**Ram Podicheti**

USA

**Laila Poisson**

USA

**Faruk Polat**

Turkey

**Zvonimir Poljak**

Canada

**Eric Polley**

USA

**Shawn Polson**

USA

**Julia Ponomarenko**

USA

**Beatriz Pontes**

Spain

**Yann Ponty**

France

**Mihai Pop**

USA

**Ryan Poplin**

USA

**Tatiana Popova**

Germany

**Robert Powers**

USA

**Shyam Prabhakar**

Singapore

**Rajesh Prasad**

Nigeria

**Morgan Price**

USA

**Eva María Priego**

Spain

**Theo Prins**

Netherlands

**Yuri Pritykin**

USA

**Melissa Pronold**

USA

**Paolo Provero**

Italy

**Fotis Psomopoulos**

Greece

**Marco Punta**

UK

**Tal Pupko**

Israel

**Sampo Pyysalo**

Japan

**Xiaoquan Qi**

UK

**Zhao-Hui Qi**

China

**Xiaoning Qian**

USA

**Huaizhen Qin**

USA

**Zhaohui Qin**

USA

**Weiliang Qiu**

USA

**Long Qu**

USA

**Lei Qu**

China

**Ronald Quinn**

Australia

**Predrag Radivojac**

USA

**Mark Ragan**

Australia

**Gajendra Raghava**

India

**Emanuele Raineri**

Spain

**Sakthivel Ramasamy**

India

**Claudia Rangel-Escareno**

Mexico

**Mattias Rantalainen**

Sweden

**Arvind Rao**

USA

**Kristoffer Rapacki**

Denmark

**Franck Rapaport**

USA

**Hooman Rashidi**

USA

**Sujeevan Ratnasingham**

Canada

**Andreas Raue**

Germany

**Tobias Rausch**

Germany

**William Ray**

USA

**Xavier Raynaud**

France

**Simon Rayner**

China

**Dietrich Rebholz-Schuhmann**

Switzerland

**Benjamin Redelings**

USA

**Elliott Rees**

UK

**Hubert Rehrauer**

Switzerland

**Jaques Reifman**

USA

**Mark Reimers**

USA

**Bernhard Renard**

Germany

**Yakir Reshef**

USA

**Boris Reva**

USA

**Federico Rey**

USA

**Alejandro Reyes**

Germany

**Andre Ribeiro**

Finland

**William Ricke**

USA

**Fabrizio Riguzzi**

Italy

**Fabio Rinaldi**

Switzerland

**Thomas Rindflesch**

USA

**Davide Risso**

USA

**Matthew Ritchie**

Australia

**Scott Ritchie**

Australia

**William Ritchie**

Australia

**Anna Ritz**

USA

**Alberto Riva**

USA

**Paul Robbins**

USA

**Christèle Robert-Granie**

France

**Stephane Robin**

France

**Marc Robinson-Rechavi**

Switzerland

**Xavier Roca**

Singapore

**Philippe Rocca-Serra**

UK

**Isabel Rocha**

Portugal

**Miguel Rocha**

Portugal

**Benjamin Roche**

France

**Thomas Rodgers**

UK

**Andrei Rodin**

USA

**Allen Rodrigo**

USA

**Christophe Roeder**

USA

**Simon Rogers**

UK

**Torbjorn Rognes**

Norway

**Igor Rogozin**

USA

**Forest Rohwer**

USA

**Judith Rollinger**

Austria

**Simona E. Rombo**

Italy

**Chiara Romualdi**

Italy

**Nancy Roosens**

Belgium

**Timo Ropinski**

Sweden

**Gail Rosen**

USA

**Paul Rosen**

USA

**Melissa Rotunno**

USA

**Joy Roy**

India

**Snehashis Roy**

USA

**Jean-Francois Rual**

USA

**Jianhua Ruan**

USA

**Cristina Rubio-Escudero**

Spain

**J. Graham Ruby**

USA

**Oscar Rueda**

UK

**Etienne Ruppe**

France

**David Russell**

USA

**Joeri Ruyssinck**

Belgium

**Colm Ryan**

Ireland

**Martin Ryberg**

Sweden

**Marc Ryser**

USA

**Achuthsankar S Nair**

India

**Ravi Sachidanandam**

USA

**Karen Sachs**

USA

**Yvan Saeys**

Belgium

**Julio Saez-Rodriguez**

UK

**Goutam Sahana**

Denmark

**Arun Sahayadhas**

India

**Satya Sahoo**

USA

**Leonidas Salichos**

USA

**Leena Salmela**

Finland

**Jared Sampson**

USA

**Selvaraj Samuel**

India

**Matthias Samwald**

Austria

**Richard Samworth**

UK

**Vartul Sangal**

UK

**Guido Sanguinetti**

UK

**Kris Sankaran**

USA

**David Sankoff**

Canada

**Daniele Santoni**

Italy

**José Santos**

Spain

**Yadav Sapkota**

Australia

**Mihaela Sardiu**

USA

**Indra Neil Sarkar**

USA

**Maureen Sartor**

USA

**Kengo Sato**

Japan

**Elke Schaper**

Switzerland

**Todd Scheetz**

USA

**Jeremy Scheff**

USA

**André Scherag**

Germany

**Thomas Schlitt**

Switzerland

**Patrick Schloss**

USA

**Bertil Schmidt**

Singapore

**Dina Schneidman**

USA

**Steffen Schober**

Germany

**Ingrid Scholtens**

Netherlands

**Dietmar Schomburg**

Germany

**Falk Schreiber**

Germany

**Lynn Schriml**

USA

**Martijn Schuemie**

Netherlands

**Rainer Schuhmacher**

Austria

**Marcel H. Schulz**

Germany

**Petra Schwalie**

UK

**Russell Schwartz**

USA

**Torsten Schwede**

Switzerland

**Simone Sciabola**

USA

**Nicola Segata**

Italy

**Lorenzo Segovia**

Mexico

**Victor Segura**

Spain

**Joan Segura Mora**

Spain

**Michael Seidl**

Netherlands

**Michael Seifert**

Germany

**Joachim Selbig**

Germany

**Chaok Seok**

South Korea

**Ilya Serebriiskii**

USA

**David Serre**

USA

**Bertrand Servin**

France

**Sohan Seth**

Finland

**Anurag Sethi**

USA

**Osman Ugur Sezerman**

Turkey

**Jahangheer Shaik**

USA

**Bruce Shapiro**

USA

**Cody Sheik**

USA

**Hong-Bin Shen**

China

**Li Shen**

USA

**Yao Shen**

USA

**Yufeng Shen**

USA

**Quanhu Sheng**

USA

**Qinghua Shi**

China

**Wei Shi**

Australia

**Xingjie Shi**

China

**Tetsuo Shibuya**

Japan

**Denis Shields**

Ireland

**Daichi Shigemizu**

Japan

**Kosaku Shinoda**

USA

**Shin-Han Shiu**

USA

**Jonas Andreas Sibbesen**

Denmark

**Thomas Sicheritz-Ponten**

Denmark

**Fabian Sievers**

Ireland

**Carlos Simmerling**

USA

**Thomas Simonson**

France

**Jared Simpson**

Canada

**David Sims**

UK

**Suzanne Sindi**

USA

**Rahul Singh**

USA

**Vijendra Singh**

India

**Jouni Sirén**

Chile

**Karthikeyan Sivaraman**

India

**Connor Skennerton**

USA

**Robert Sladek**

Canada

**Joseph Slagel**

USA

**Marek Sliwinski**

USA

**Rafael Slodzinski**

Germany

**Colin Smith**

Germany

**Gordon Smyth**

Australia

**Graham Snyder**

USA

**Johannes Soding**

Germany

**Charlotte Soneson**

Switzerland

**Yangqiu Song**

USA

**Rainer Spang**

Germany

**Jana Sperschneider**

Australia

**Josef Spidlen**

Canada

**Narayanaswamy Srinivasan**

India

**Tharuvai Sriram**

USA

**Mugdha Srivastava**

Germany

**Efstathios Stamatatos**

Greece

**Mario Stanke**

Germany

**Alexander Statnikov**

USA

**Oliver Stegle**

UK

**Amelie Stein**

USA

**Ulrich Stelzl**

Germany

**Brian Stevenson**

USA

**Edward Stites**

USA

**Glenn Stone**

Australia

**Marc Streit**

Austria

**Blaz Stres**

Slovenia

**Torsten Struck**

Germany

**Amber Stubbs**

USA

**Henk Stunnenberg**

Netherlands

**Emily Chia-Yu Su**

Taiwan

**Gang Su**

USA

**Zhen Su**

China

**Masahiro Sugimoto**

Japan

**Fengzhu Sun**

USA

**Hao Sun**

Hong Kong

**Qi Sun**

USA

**Shuying Sun**

USA

**Zhifu Sun**

USA

**Yanni Sun**

USA

**Yutaka Suzuki**

Japan

**Neil Swainston**

UK

**S Joshua Swamidass**

USA

**Firas Swidan**

Israel

**Aniko Szabo**

USA

**Sing-Hoi Sze**

USA

**David Tabb**

USA

**Fumihiko Takeuchi**

Japan

**Naoko Takezaki**

Japan

**Koichiro Tamura**

Japan

**Yuande Tan**

USA

**Haixu Tang**

USA

**Wei Tang**

USA

**Miljana Tanic**

Serbia

**Eric Tannier**

France

**Xiaohui Tao**

Australia

**Luis Tari**

USA

**Tolga Tasdizen**

USA

**Cenny Taslim**

USA

**Stefanie Tauber**

Germany

**Simon Tavaré**

UK

**Ali Tavassoli**

UK

**James Taylor**

USA

**Michel Tchuenche**

Tanzania

**Sebastien Tempel**

France

**Michaela Teravest**

USA

**Tyler Thacker**

USA

**Jitendra Thakur**

India

**Margarita Theodoropoulou**

Greece

**Nathalie Théret**

France

**Andreas Tholey**

Germany

**Sean Thomas**

USA

**Xiangjun Tian**

USA

**Elisabeth Tillier**

Canada

**Alexandru Tomescu**

Finland

**Kentaro Tomii**

Japan

**Stefano Toppo**

Italy

**Andrew Torda**

Australia

**Manabu Torii**

USA

**Petri Toronen**

Finland

**Silvio Tosatto**

Italy

**Jorg Tost**

France

**Zlatko Trajanoski**

Austria

**Mark Transtrum**

USA

**Todd J Treangen**

USA

**Edward Trifonov**

Israel

**William Trimble**

USA

**Urmi Trivedi**

UK

**Ioannis Tsamardinos**

Greece

**George Tseng**

USA

**Yoshimasa Tsuruoka**

UK

**Catalina Tudor**

USA

**Pierre Tuffery**

France

**Nurcan Tuncbag**

Turkey

**Nicolas Turenne**

France

**Asuman Turkmen**

USA

**Ernest Turro**

UK

**Susan Tweedie**

UK

**Hiroki R Ueda**

Japan

**Masao Ueki**

Japan

**Snekhalatha Umapathy**

India

**Philip Uren**

USA

**Filippo Utro**

USA

**Iosif Vaisman**

USA

**Sandor Vajda**

USA

**Ilya Vakser**

USA

**Giorgio Valentini**

Italy

**Giorgio Valle**

Italy

**Diana Valverde**

Spain

**Stefan Van Aelst**

Belgium

**Mark Van De Wiel**

Netherlands

**Jeroen Van Dijk**

Netherlands

**Jan Van Oeveren**

Netherlands

**Saran Vardhanabhuti**

USA

**Sudhir Varma**

USA

**Gilberto Vaughan**

USA

**Gert Jan Veenstra**

Netherlands

**Narayanan Veeraraghavan**

USA

**Karthikraja Velmurugan**

India

**Kasinadar Veluraja**

India

**Ricardo Vencio**

Brazil

**Lieven Verbeke**

Belgium

**Ricardo Verdugo**

Chile

**Stella Veretnik**

USA

**Linda Verhoef**

Netherlands

**Karin Verspoor**

Australia

**Jean-Philippe Vert**

France

**Wynand Verwoerd**

New Zealand

**Jean-Baptiste Veyrieras**

France

**Francesco Vezzi**

Sweden

**Saverio Vicario**

Italy

**Arunachalam Vinayagam**

USA

**Francisco Vital-Lopez**

USA

**Olga Vitek**

USA

**Ioannis Vlachos**

Greece

**Horatiu Voicu**

USA

**Benjamin Voight**

USA

**Thomas Vondriska**

USA

**Lane Votapka**

USA

**Thom Vreven**

USA

**Gert Vriend**

Netherlands

**Matthew Wakefield**

Australia

**Jerome Waldispuhl**

Canada

**Levi Waldron**

USA

**Byron Wallace**

USA

**Bjorn Wallner**

Sweden

**W. David Walter**

USA

**Dirk Walther**

Germany

**Xiang Wan**

Hong Kong

**Ilona Wandzik**

Poland

**Dennis Wang**

Canada

**Guangshun Wang**

USA

**Hong Wang**

USA

**Huaichun Wang**

Canada

**Jing-Fang Wang**

China

**Jing Wang**

USA

**Junbai Wang**

Norway

**Jianxin Wang**

China

**Ming Wang**

USA

**Ying Wang**

USA

**Shengzhi Wang**

USA

**Jiguang Wang**

USA

**Liya Wang**

USA

**Xueqin Wang**

China

**Yu-Ping Wang**

USA

**Yc Wang**

China

**Zhong Wang**

USA

**Zhanyong Wang**

USA

**Tandy Warnow**

USA

**Andrew Warren**

USA

**Rene Warren**

Canada

**Gunther Weber**

USA

**Lawrence Wee**

Singapore

**Martin Weigt**

France

**William Weir**

USA

**Michael Wendl**

USA

**Michel Westenberg**

Netherlands

**Sebastian Will**

USA

**Clarlynda Williams-Devane**

USA

**Stacey Winham**

USA

**Anthony Wirth**

Australia

**Michael J. Wise**

Australia

**Gerd Wohlfahrt**

Finland

**Kyoung Jae Won**

USA

**Gane Ka-Shu Wong**

Canada

**Garry Wong**

Finland

**Jason Wong**

UK

**King-Fung Wong**

Australia

**Chung Wong**

USA

**Jun Woo**

USA

**Derrick Wood**

USA

**Jonathan Woodsmith**

Germany

**Liz Worthey**

USA

**Gavin Wright**

UK

**Fang-Xiang Wu**

Canada

**Hao Wu**

USA

**Shuang Wu**

USA

**Song Wu**

USA

**Stephen Wu**

USA

**Cen Wu**

USA

**Ming Wu**

USA

**Xuebing Wu**

USA

**Zuowei Wu**

USA

**Yu-Wei Wu**

USA

**Li Xia**

USA

**Zheng Xia**

USA

**Jingfa Xiao**

China

**Xuan Xiao**

China

**Yi Xiao**

China

**Lei Xie**

USA

**Hongyi Xin**

USA

**Fuyong Xing**

USA

**Dong Xu**

USA

**Rong Xu**

USA

**Wei Xu**

Canada

**Yanxun Xu**

USA

**Jianhua Xuan**

USA

**Zhenyu Xuan**

USA

**Yu Xue**

China

**Atsuko Yamaguchi**

Japan

**Yasunori Yamamoto**

Japan

**Can Yang**

USA

**Yang Yang**

Japan

**Jialiang Yang**

USA

**Jean Yang**

Australia

**Lin Yang**

USA

**Jinn-Moon Yang**

Taiwan

**Runqing Yang**

China

**Wen-Yun Yang**

USA

**Xinan Yang**

China

**Jianyi Yang**

USA

**Xuerui Yang**

China

**Zhihao Yang**

China

**Yuedong Yang**

Australia

**Youngik Yang**

USA

**Yuan Yao**

USA

**Stephen Yau**

USA

**Omer Nebil Yaveroglu**

USA

**Ning Ye**

USA

**Yuzhen Ye**

USA

**Meliha Yetisgen-Yildiz**

USA

**Huangdi Yi**

China

**Kevin Yip**

Hong Kong

**Byung-Jun Yoon**

USA

**Chenggang Yu**

USA

**Fuli Yu**

USA

**Fengchao Yu**

Hong Kong

**Weimiao Yu**

Singapore

**Yang Yu**

USA

**Zu-Guo Yu**

China

**Yinyin Yuan**

UK

**Elizabeth Yuriev**

Australia

**Martin Zacharias**

Germany

**Lan Zagar**

Slovenia

**Dirk Zajonc**

USA

**Federico Zambelli**

Italy

**Mihaela Zavolan**

Switzerland

**Haimao Zhan**

USA

**Xianquan Zhan**

China

**Aidong Zhang**

USA

**Bo Zhang**

USA

**Chaolin Zhang**

USA

**Han Zhang**

USA

**Henry Zhang**

USA

**Jian Zhang**

USA

**Jing Zhang**

USA

**Kurt Zhang**

USA

**Li Zhang**

USA

**Louxin Zhang**

Singapore

**Qiangfeng Zhang**

USA

**Shu-Dong Zhang**

UK

**Shuxing Zhang**

USA

**Xiuwei Zhang**

UK

**Shaowu Zhang**

China

**Tong Zhang**

Hong Kong

**Yusen Zhang**

China

**Zhang Zhang**

China

**Guoyan Zhao**

USA

**Hongyu Zhao**

USA

**Huiying Zhao**

Australia

**Hao Zhao**

USA

**Xingming Zhao**

China

**Fangqing Zhao**

China

**Ding Zhaohua**

USA

**Yan Zhen**

China

**Chunfang Zheng**

Canada

**Heping Zheng**

USA

**Jie Zheng**

USA

**Siyuan Zheng**

USA

**Xiaobin Zheng**

USA

**Shan Zhong**

USA

**Du Zhou**

China

**Beiyan Zhou**

USA

**Qiangjun Zhou**

USA

**Tianyin Zhou**

USA

**Tong Zhou**

USA

**Wenjin Zhou**

USA

**Wanding Zhou**

USA

**Xiaobo Zhou**

USA

**Yaoqi Zhou**

USA

**Jianlong Zhou**

Australia

**Hufeng Zhou**

USA

**Dongxiao Zhu**

USA

**Jun Zhu**

USA

**Ruoqing Zhu**

USA

**Wensheng Zhu**

China

**Bin Zhu**

USA

**Yunping Zhu**

China

**Ralf Zimmer**

Germany

**Quan Zou**

China

**Marcel Zwietering**

Netherlands

